# Supplementary material for: Structural plasticity for neuromorphic networks with electropolymerized dendritic PEDOT connections
Source: Nat Commun. 2023 Dec 8;14:8143. doi: 10.1038/s41467-023-43887-8 (PMC10709651; doi:10.1038/s41467-023-43887-8)
Supplement: Supplementary file 1 — supplementary informations [file 41467_2023_43887_MOESM1_ESM.pdf]

A Supplementary information

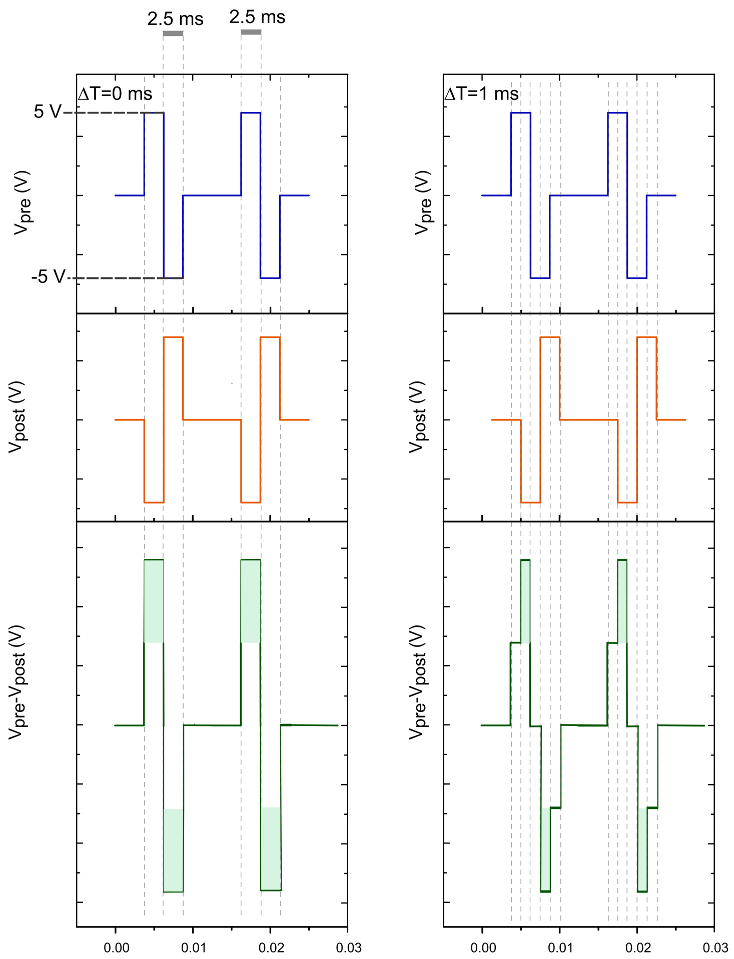

Supp. Figure 1: Correlation reproduced through pre- and post-signals timing. As a result of potential difference between electrodes ($V_{\text{pre}} - V_{\text{post}}$), effective voltage overlap is formed (green curve). Overpotentiation takes place (green highlighted regions) and results in electropolymerization. At $\Delta T = 0$ ms longer over potential duration occurs which is related to strong correlation while at $\Delta T = 1$ ms overpotential duration is reduced and corresponds to lower correlation. Note that pre- and post-pulses are opposite in polarity.

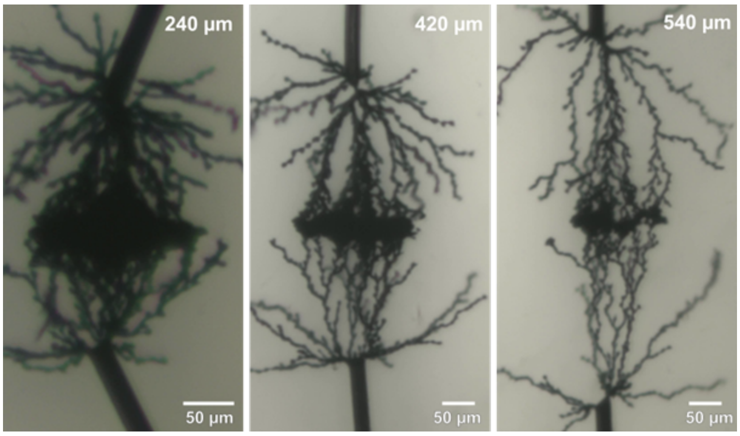

Supp. Figure 2: Effect of distance. Optical microscope images of dendritic morphologies synthesized at different spacing between electrodes. Electropolymerization was carried out at distances from 240 µm to 540 µm. Larger distances were not tested due to technical limitations of the setup.

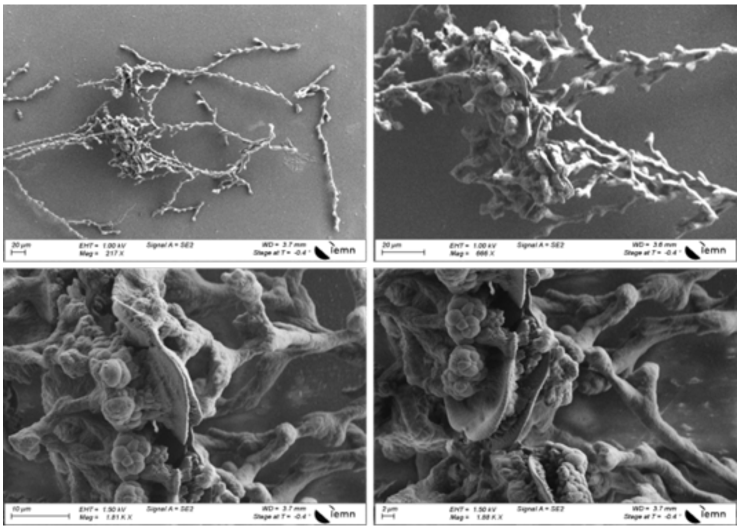

Supp. Figure 3: Dendrites morphology. Scanning electron microscope images of polymer dendritic structure (grown at 540µm distance) after its extraction from the electrolyte.

		**Frequency**		
		**130 Hz**	**80 Hz**	**20 Hz**
**ΔT**	**0 ms**	148 s	180 s	612 s
	**0.5 ms**	150 s	300 s	614 s
	**1.0 ms**	124 s	150 s	667 s
	**1.5 ms**	122 s	151 s	0 s
	**2.0 ms**	138 s	173 s	0 s

Supplementary Table 1: Completion time extracted for the different experiments in Fig. 2.

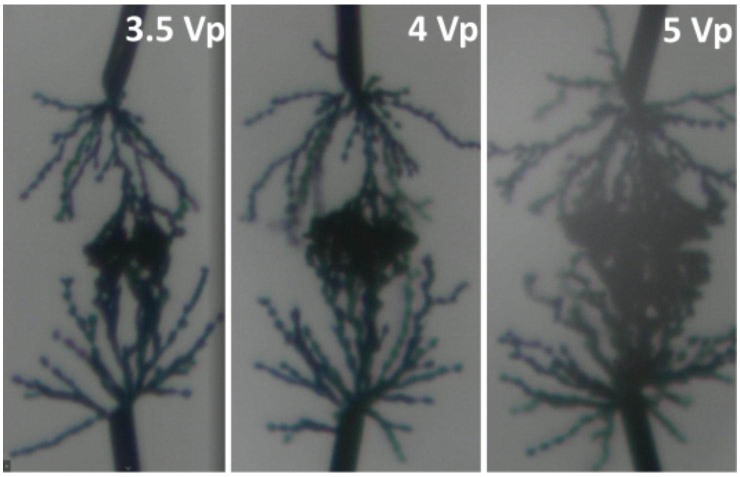

Supp. Figure 4: Effect of applied voltage on dendritic morphology. Optical images of dendritic branches developed via STDP mechanism at constant distance 240µm, fixed frequency $f_{\mathrm{pre}} = f_{\mathrm{post}} = 80$ Hz, $\Delta T = 0$ ms and various voltage amplitude V+/V-: 3.5 V_p/-3.5 V_p, 4 V_p/-4 V_p and 5V_p/-5V_p. Images were taken 30s after first connection in between branches.

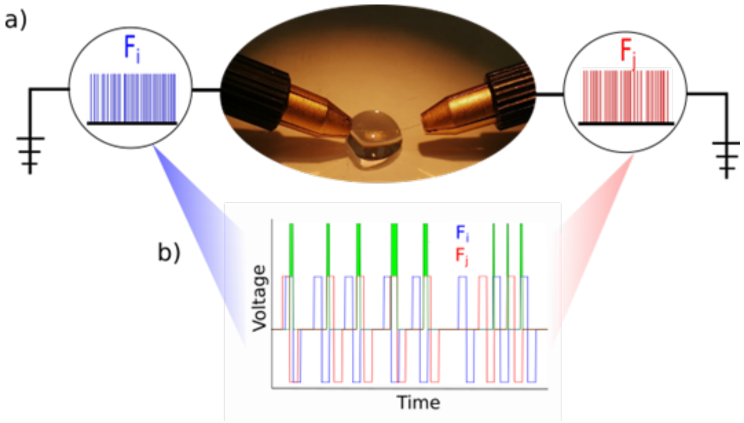

Supp. Figure 5: SRDP pulse correlation. For SRDP implementation, identical bipolar pulses of 0.6 ms width and 10 $V_{\text{peak-to-peak}}$ were applied from both terminal. Note that pre- and post-neurons don't need to be specified since signals are identical. Pulse duration was adapted to ensure a range of frequency modulation in between 0 and 1000 Hz.

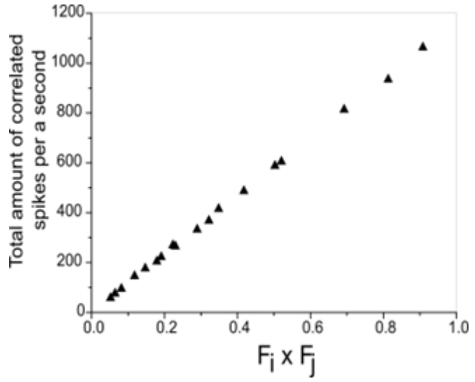

Supp. Figure 6: Total amount of correlated spikes per second resulting from pulses' overlapping when two Poisson-like spike trains at mean frequency F_i and F_j are applied from the two terminals.

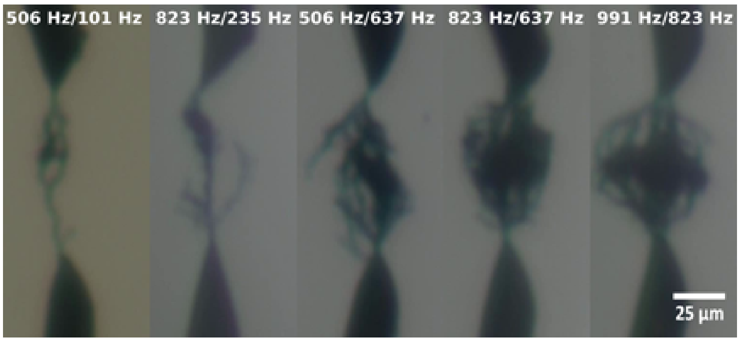

Supp. Figure 7: Dendritic morphologies developed through utilization of SRDP signals. Microscopic images of five different dendrites obtained by pairing bipolar pulses with different frequency values. Images were taken 30s after connection in between branches.

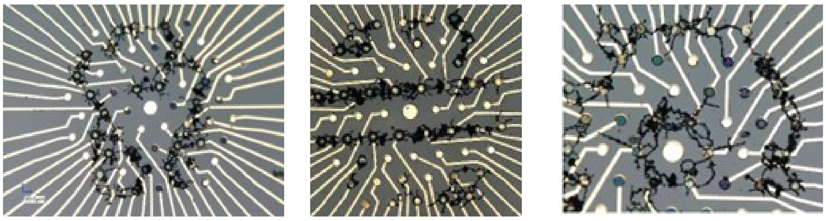

Supp. Figure 8: Example of dendritic growth on 2D microelectrode arrays. Dendritic connections in between nodes were grown sequentially and illustrate a possible hardware implementation of structural plasticity.

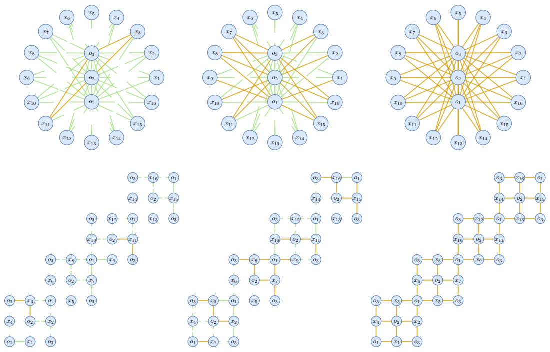

Supp. Figure 9: Example of hardware mapping of the classification task in Fig. 5 on a 2D network of nodes. Note that each node can only connect with its closest neighbors, thus ensuring no dendrites overlap and connection mismatch.

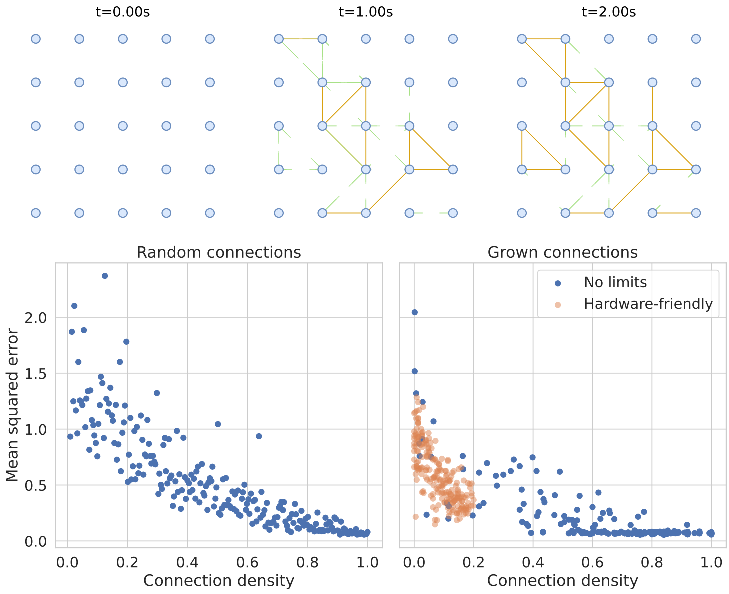

Supp. Figure 10: Additional hardware constraints included in the auto-encoding task. In the hardware-friendly case, connections can only occur in between neighboring nodes. The mean square error for the hardware constrained networks is represented in orange. The same trend in finding the optimal topology with structural plasticity is observed. Note that the connection density saturate in this example due to limited possibility of connections. The number of connections could be increased by increasing the number of nodes (i.e. from a 5×5 mesh network to a N×N network).

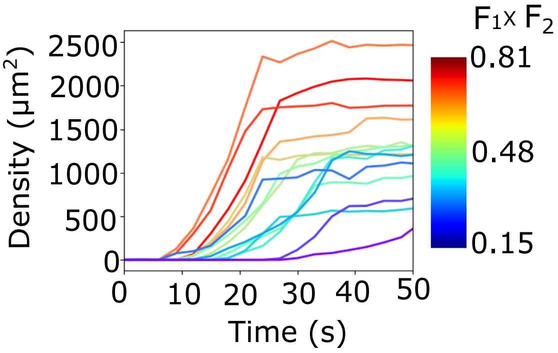

Supp. Figure 11: Evolution of the effective dendrite area. The dendrites are projected on a 2D image and analyzed using ImageJ software. During electropolymerization with bipolar pulses, we observed that after touching and forming connection between each other, the dendrites stop their growth and experienced only minimal modifications. This effect could be explained by a redistribution of the voltage potential drop in the system electrodes/dendrites/electrolyte when the dendrites connect each other. We can see that after a sublinear regime (the dendritic growth) each experiment saturate to a fix density (growth completion). We used the onset of saturation to define the completion time and to evaluate the conductance of the dendrites.

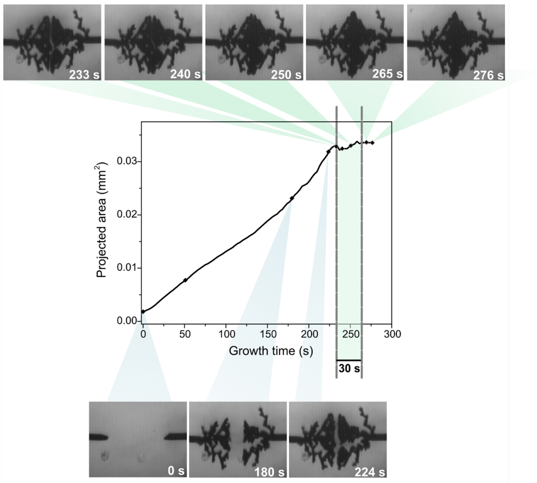

Supp. Figure 12: Projected area over time for dendrites grown at Vp = 5 V (Voff = 0 V, f = 80 Hz, dc = 50%) with microscopic images showing dendrites' changes at different growth times.

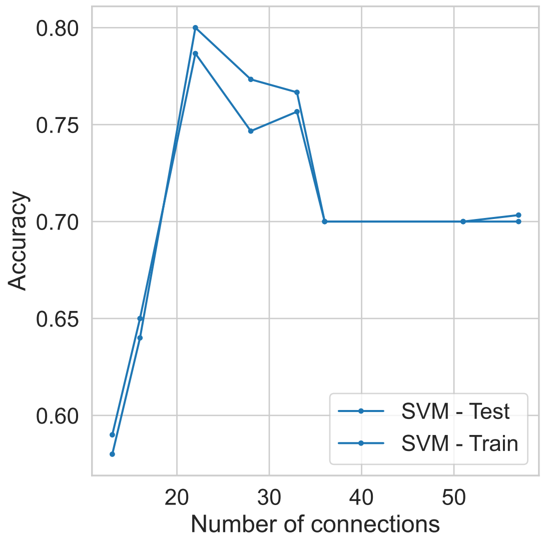

Supp. Figure 13: Evolution of the classification accuracy for both the train and test dataset. Overfitting can be evidenced when accuracy on training dataset increase or saturate while accuracy on testing dataset starts to drop. As the trend in accuracy vs connection density is similar for both the training and testing sets, the overfitting hypothesis is rejected. This highlight that an optimal sparse topology corresponding to an optimal projection of the signals exist for this task.

A.1 Structural plasticity simulation model

The structural plasticity model was simplified to simulate higher-level tasks. The physical increment of each dendrite is linearly dependent on the pulse overlap between the presynaptic and postsynaptic neurons. Each neuron emits a perfectly square pulse of duration D_{pulse}. Hence, the pulse shape can be modeled as:

$$P(t) = A(\mathcal{H}(t - t_{\text{spike}}) - \mathcal{H}(t - t_{\text{spike}} - D_{\text{pulse}})) \quad (1)$$

where $\mathcal{H}$ is the Heaviside step function, t_{spike} is the moment of a spike and A is the amplitude of the pulse. The physical increment is represented by a variable $s \in \mathcal{R}^+$. The overlap between a pulse P_{pre} and P_{post}, for the pulses of the pre- and post-synaptic neurons respectively, will grow the dendritic branch:

$$\dot{s} = P_{\text{pre}}(t) \cdot P_{\text{post}}(t) \quad (2)$$

When the variable s reaches a threshold that is dependent on the Euclidean distance between the pre- and post-neuron in a simulated physical space, a weighted synaptic connection is created between these two neurons at time t_{conn}. As the weight of the connection is also dependent on the firing frequency of both the pre- and post-neurons, two exponentially decaying traces k are added in the model of the conductance:

$$\dot{w} = \mathcal{H}(t - t_{\text{conn}}) \cdot k_{\text{pre}}(t) \cdot k_{\text{post}}(t) \cdot P_{\text{pre}}(t) \cdot P_{\text{post}}(t) \quad (3)$$

where k_{pre} and k_{post} are traces for the pre- and post-neurons respectively with $\dot{k}_i = \frac{-k}{\tau}$.

The model presented is a simplification of the behavior of the growing physical dendritic branch. As such, the constants presented (pulse duration D_{pulse}, pulse amplitude A, decaying trace constant τ and the distance between the neurons) are not representative of the physical experiment. These constants can therefore be fitted to match the software model with the physical representation. Main text, Fig. 4b-c presents the growth rate and the conductance obtained in an experimental setting compared with the model with manually fitted constants to best fit the data. In both experiments, Poisson spikes were generated at various frequencies between two simulated neurons. Higher frequencies increase the number of overlapped pulses, which decreases the time necessary to create a connection between the neurons.

A.2 Surface electromyography classification task

The structural plasticity data extracted from the software model was used to perform electromyography (EMG) classification. The Roshambo surface EMG (sEMG) dataset from [1] was used with the same signal-to-spikes encoding scheme of [2] and presented in Supp. Fig. 14. This dataset contains three classes, representing the rock, paper or scissor movements made while wearing a sEMG armband from 10 participants with eight electrodes. Every channel is converted to spike by first up-sampling the signal to 1000 Hz and then comparing the second discrete difference with an up or down threshold representing two spike train inputs per channel of the signal. The signal to spikes methodology employed is further described in [2]. The first and last 600ms of each 2s sample were trimmed-off.

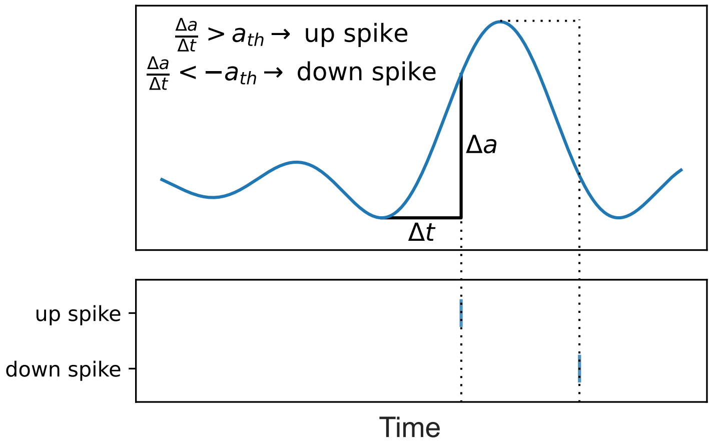

Supp. Figure 14: Schematic representation of the spike encoding scheme. When the relative amplitude of the signal $\frac{\Delta a}{\Delta t}$ reaches the threshold a_{th} ($-a_{\mathrm{th}}$) an up (down) spike is emitted.

A connectivity matrix is created using the 16 spiking inputs and three spiking output neurons. The output neurons are set to randomly spike based on a Poisson distribution when the label matches the current sample. Each sample of the dataset is fed to the network, and the time for each pair of connection to form is recorded. Note that in this benchmark, the connections are created individually without influence from previously generated connections. Given enough time, the connectivity matrix will be fully connected as there is no detrimental effect in the growing dendrite model. As such, the model with the connectivity matrix at different points in time is tested to compare connectivity density vs performance. The connectivity density is equal to the number of connections at a certain point in time divided by the maximum number of connection possible.

Two different training strategies are compared using this generated connectivity matrix in the three following subsections. The network topology associated with each training strategy is presented in main text, Fig.5b. In all the strategies, the third session of the EMG dataset is used as a test set, and the first and second sessions are used as a training set. The final accuracy results of each method are presented in main text, Fig.5d as a function of the connection density.

A.2.1 Support Vector Machine

The first strategy is to use a linear Support Vector Machine (SVM) connected to the output of the three classification neurons. In this scheme, the grown connections between the 16 input channels of the EMG and the three classification neurons are used unweighted. The spikes are counted based on the connectivity to each input neurons for classification. The spike counts are normalized by removing the mean and scaling to unit variance for each feature, with the statistical measures computed from the training set, before being fed to the SVM with a regularization parameter of one.

A.2.2 Stochastic Gradient Descent

The second strategy consists of using the sparse connectivity matrix created from the dendritic model as the sole layer of the network. Like the first strategy, the spikes are counted for classification and normalized with the mean and variance of each feature of the training set. Unlike the first strategy, the spikes are counted directly at the input layer, representing 16 spike counts. The correlation between each pre- and post-neurons, as given by the inverse of the time to grow the dendrite, is multiplied to the weight between the neurons as such:

$$u_j = b_j + \sum_i x_i \cdot w_{ij} \cdot c_{ij} \tag{4}$$

where u_j is the state of the output neuron j before the activation function, x_i is the normalized spike count of each neuron i, w_{ij} and c_{ij} are the weight and correlation coefficient between each neuron i and output neuron j and b_j is a bias for neuron j. The training sampled are batches with 64 samples per batch, and the cross-entropy loss is computed for each batch. The network is optimized with MADGRAD [3] with learning rate of 0.01 for up to 5k epochs.

A.3 Sparse unsupervised spike auto-encoding task

A recurrent network of spiking leaky-integrate-and-fire (LIF) neurons was created to encode signals following the work of [4]. In their work, the recurrent network was either fully connected or randomly connected with various target connectivity densities. Here, the connectivity matrix is first defined using structural plasticity and then the model is trained to encode signals with spikes, as done in [4]. The structure of the network is presented in main text, Fig.5a. First, a signal of random white noise is generated for two seconds with two independent channels. This analog signal is fed to the network using sparse localized connectivity from the two channels to 25 LIF neurons as weighted input currents. Each input channel is connected to three random neurons from the recurrent pool and its close neighbors, similar to a branching dendrite. The input connectivity is fixed, along with the input weights, during the whole simulation. The structural plasticity model is run to create all the recurrent connections, except the self-connections, which are used as a reset mechanism for the LIF neurons and are always strongly inhibitory. The neurons are placed in a two-dimensional lattice graph, and each neuron is distanced equally between each non-diagonal neighbors. Once a connection is created, a random initial weight is associated with the new connection that can be either positive or negative. Each new connection contributes to the dynamic of the network and the resulting network activity is further used to create connections iteratively for the entirety of the signal. The direction of newly created connection depends on the neuron whose spike allowed the growth to reach the other neuron. Connections will almost always be bi-directional as correlation dictates growth and the inverse relation is equivalent. Once the connectivity matrix has been established, a new two-channel signal of 50 seconds is generated. For each of the created connection, a simplified version of the plasticity rule of [4] is added, and the network learns the synaptic weight for each connection. The plasticity rule is given by the following equation:

$$\dot{w} = -\alpha(\beta \cdot u_{post} + w + \mu)\delta(t - t_{pre}) \tag{5}$$

with α, β and μ representing chosen constants, u_{post} the membrane potential of the postsynaptic neuron and t_{pre} the time of a presynaptic spike. δ is the Dirac function, numerically equal to one when $t = t_{\text{pre}}$ and zero otherwise. This function was derived in [4] to enhance the encoding capability of a recurrent network and to maximize sparsity, which we simplified slightly without losing in encoding efficiency. Once the weights have converged, after the entirety of the training 50 seconds signal, a decoding matrix is computed to reduce the least square error between the exponentially filtered spike output of the reservoir and the original signal. Finally, all the weights are frozen, and a new ten-second signal is generated for testing purposes. The final encoding mean squared error is computed using the test signal. We conducted the same experiments with various structural plasticity parameters (pulse duration, interneuron distance), which yielded various connection densities and encoding performances. For comparison, we tested the same approach but using random connections instead of using structural plasticity. The performance of each of these networks is reported in main text, Fig. 6c.

References

[1] Elisa Donati. *EMG from forearm datasets for hand gestures recognition.* May 2019. DOI: 10.5281/zenodo.3194792.

[2] Nikhil Garg et al. "Signals to Spikes for Neuromorphic Regulated Reservoir Computing and EMG Hand Gesture Recognition". In: *International Conference on Neuromorphic Systems 2021.* ICONS 2021. Knoxville, TN, USA: Association for Computing Machinery, July 2021, pp. 1–8. DOI: 10.1145/3477145.3477267.

[3] Aaron Defazio and Samy Jelassi. "Adaptivity without Compromise: A Momentumized, Adaptive, Dual Averaged Gradient Method for Stochastic Optimization". In: (Jan. 2021).

[4] Wieland Brendel et al. "Learning to represent signals spike by spike". en. In: *PLoS computational biology* 16.3 (Mar. 2020), e1007692. DOI: 10.1371/journal.pcbi.1007692.
