# Supplementary material for: Structural plasticity for neuromorphic networks with electropolymerized dendritic PEDOT connections
Source: Nat Commun. 2023 Dec 8;14:8143. doi: 10.1038/s41467-023-43887-8 (PMC10709651; doi:10.1038/s41467-023-43887-8)
Supplement: Supplementary file 3 — Description of Additional Supplementary Files [file 41467_2023_43887_MOESM3_ESM.docx]

**Description of Additional Supplementary Files**

**File Name: Supplementary Movie 1
Description:** Optical video of the dendritic growth with applied pulsed voltage. The growth is a result of the STDP mechanism at constant distance, fixed frequency fpre = fpost = 80 Hz, ΔT=1ms and a voltage amplitude V+/V- of 5V/-5V.
